# Accurate and Rapid Identification of Longan Arillus and Litchi Semen by a Multiplex PCR Assay

**DOI:** 10.3390/plants9080948

**Published:** 2020-07-28

**Authors:** Wook Jin Kim, Sungyu Yang, Goya Choi, Inkyu Park, Pureum Noh, Min Jee Kim, Byeong Cheol Moon

**Affiliations:** Herbal Medicine Resources Research Center, Korea Institute of Oriental Medicine, Daejeon 34054, Korea; ukgene@kiom.re.kr (W.J.K.); sgyang81@kiom.re.kr (S.Y.); serparas@kiom.re.kr (G.C.); pik6885@kiom.re.kr (I.P.); pureum322@kiom.re.kr (P.N.); minjeekim3@kiom.re.kr (M.J.K.)

**Keywords:** Longan, Litchi, Rambutan, multiplex PCR assay, herbal medicine

## Abstract

*Dimocarpus longan*, *Litchi chinensis*, and *Nephelium lappaceum* are commercially valuable subtropical and tropical fruits of the Sapindaceae family. Arillus and seeds of the three species have very similar morphologies; however, the arillus of *D. longan* is used as the herbal medicine Longan Arillus and seeds of *L. chinensis* are used as Litchi Semen in Korean and Chinese pharmacopoeias. The adulteration of herbal medicines with inauthentic species, including the use of Aril and seed fractions acquired from a single species for two herbal medicines (e.g., Longan Arillus and Litchi Semen), is often driven by economic motives. DNA markers are a tool for the detection of adulterants in commercial products. To establish rapid and reliable assays for the genetic identification of authentic Longan Arillus and Litchi Semen, we developed DNA markers with high specificity and sensitivity based on internal transcribed spacer (ITS) sequences. The newly developed DNA markers and multiplex PCR assay may contribute to efforts to protect against adulteration, quality control, and the standardization of herbal medicines.

## 1. Introduction

Longan (*Dimocarpus longan* Lour.), litchi (*Litchi chinensis* Sonn.), and rambutan (*Nephelium lappaceum* L.) are tropical and subtropical fruits belonging to the family Sapindaceae, which includes 1900 species and 144 genera [1,2,3]. These fruits are popular in South Korea, but the climate of South Korea is not appropriate for growth owing to its geographical position in a temperate zone. Major exporting countries are China, India, Sri Lanka, Thailand, and Malaysia. In addition, longan and litchi are the sources of the herbal medicines ‘Longan Arillus’ and ‘Litchi Semen,’ respectively, defined in Korean pharmacopoeias (KP) and Chinese pharmacopoeias (ChP), and they are food materials permitted in the Korean Food Standards Codex (KFSC) [4]. Rambutan is permitted for use as a food material in KFSC, but not for herbal medicines in KP or CP. Longan Arillus is used to treat anxiety and insomnia, and it has a wide range of beneficial pharmacological effects, including antioxidant, anxiolytic, vasorelaxation, and anticancer activity [5]. Litchi Semen is used to treat colds and pain [6]. Therefore, the two herbal medicines have distinct applications. Nevertheless, the economically motivated adulteration of herbal medicines with inauthentic materials is a major issue [7,8]. Fruits of these three species have different morphologies, but the arillus and seeds from the peeled fruits are very similar. It is possible to produce two herbal medicines from the fruits of one species. Adulterants are common in the Korean herbal market; however, studies of authentic and closely related species are lacking.

DNA barcoding analyses are widely used for species identification in plants (based on internal transcribed spacer [ITS], *mat*K, and *rbc*L), animals (COI), fungi (ITS), and microorganisms (ITS) [9,10,11]. Most herbal medicines are derived from plant taxa. The ITS region has an advantage over *mat*K and *rbc*L with respect to species identification owing to the high genetic differentiation between closely related species [10]. Nevertheless, DNA barcoding analyses require particular conditions for accurate results, including precise sample conditions to minimize DNA damage and the existence of a species-specific sequence [12,13]. In particular, it is difficult to analyze PCR amplification in herbal medicines produced from tropical and subtropical fruits rich in polyphenolic or polysaccharide compounds, which disrupt the isolation of intact DNA. Additionally, herbal medicines processed in a single package are distributed in large quantities, including fragmented samples. In this case, DNA barcoding analyses of individual samples are needed for species identification. Therefore, DNA analysis methods with high sensitivity are necessary for accurate species identification in herbal medicines in which mixed, broken, dried, and chemically treated samples are typical [8]. Sequence characterized amplified region (SCAR) markers have recently been applied as an alternative to DNA barcoding for analyses of herbal medicines, such as Zanthoxyli Pericarpium, Araliae Continentalis Radix, Angelicae Dahurica Radix, and Adenophorae Radix [8,9,12,14]. SCAR markers involve short species-specific sequence fragments, unlike DNA barcoding, requiring analyses of full-length target sequences.

Adulterants in herbal medicines vary in quantity from trace amounts to entire samples, and can be explained by economical motivation or unintended processing [9,15]. Therefore, DNA markers should have high sensitivity and species-specificity for applications to mixed herbal medicines. Multiplex PCR assays show powerful discriminability for the identification of more than two species simultaneously [8,16].

The aim of this study was to develop a rapid species-specific multiplex assay with high sensitivity using SCAR markers for species identification in herbal medicines marketed as Longan Arillus and Litchi Semen. The newly developed tool will be useful for the standardization and quality control of traditional herbal medicines.

## 2. Results

### 2.1. Sequence Properties and Phylogenetic Analysis

ITS sequences with four replicates were confirmed using 15 samples of three species; these sequences were confirmed using the GenBank database of the National Center for Biotechnology Information (NCBI; see Supplementary Materials Table S1).

**Figure 1:**
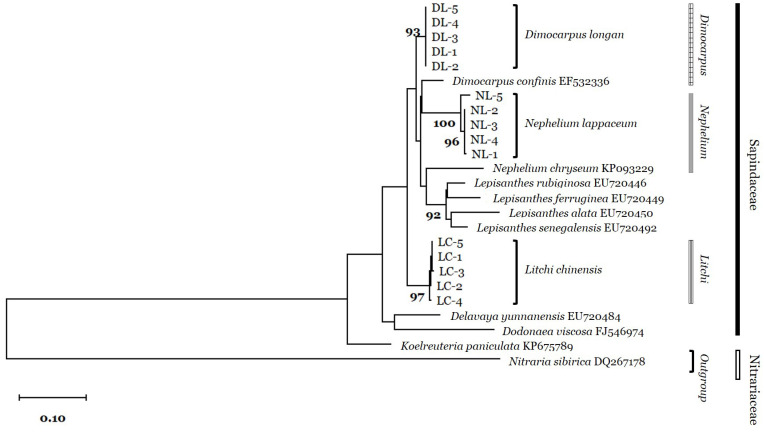
Neighbor-joining (NJ) tree based on ITS sequences for *D. longan*, *L. chinensis*, *N. lappaceum*, and nine closely related species in Sapindaceae. The NJ tree was obtained using MEGAX with 1000 bootstrap replicates. Scale bar represents 0.10 substitutions per site.

Phylogenetic relationships were analyzed by the neighbor-joining (NJ) method based on ITS sequences of the three species and nine closely related species in Sapindaceae. The three species clustered into monophyletic groups, and *D. longan* and *L. chinensis* were more closely related to each other than to *N. lappaceum* (Figure 1). Interspecific distances were 0.0925 ± 0.0179 for *D. longan*, 0.0880 ± 0.0130 for *L. chinensis*, and 0.1051 ± 0.0067 for *N. lappaceum*, indicating that *N. lappaceum* exhibits the greatest divergence, consistent with the results of the phylogenetic analysis (Table 1).

### 2.2. Singleplex and Multiplex SCAR Assays

Using ITS barcoding, we sequenced 15 samples (Supplementary Materials Figure S1) and generated an alignment of 686 bp (equal sizes for each species) for the identification of nucleotide substitutions and insertions/deletions (indels) separating the three species. Based on Interspecific differences and conserved intraspecific sequence regions, candidate primers were designed for singleplex assays as well as a multiplex PCR assay aimed at the simultaneous discrimination of species (Table 2 and Supplementary Materials Figure S1).

**Figure 2:**
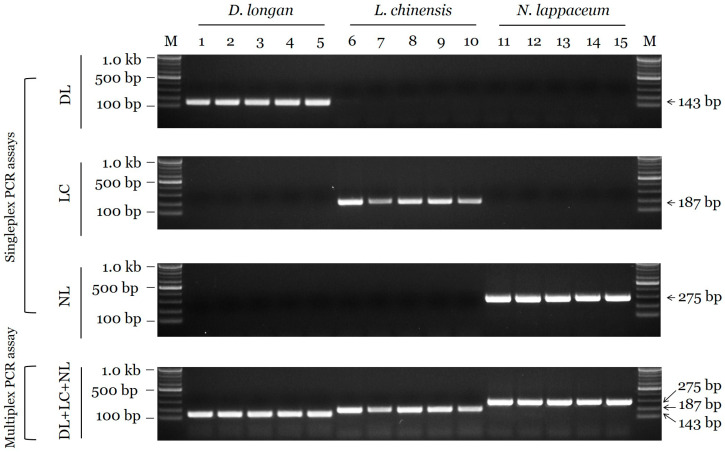
Specificity of singleplex and multiplex PCR assays for *D. longan*, *L. chinensis*, and *L. lappaceum*. M: 100 bp DNA size marker (100–1000 bp), Lanes 1–5: *D. longan*, Lanes 6–10: *L. chinensis*, Lanes 11–15: *N. lappaceum*. DL, LC, and NL indicate the primer sets. Arrowheads indicate the amplified fragment sizes.

Candidate primers were evaluated by species-specific amplification (i.e., a lack of amplification in nontarget species). The candidate primers only generated amplicons in the target species, indicating species-specificity; product sizes were 143 bp in *D. longan*, 189 bp in *L. chinensis*, and 276 bp in *N. lappaceum* (Table 2 and Figure 2). Additionally, using the multiplex PCR assay, we simultaneously discriminated between species using the species-specific SCAR markers (Figure 2).

### 2.3. Specificity and Sensitivity

The three primer sets (DL, LC, and NL), referred to as SCAR markers, were evaluated with respect to cross-reactivity against 14 plant, 8 animal, and 1 fungal species used as herbal medicines (Table 3 and Supplementary Materials Table S2). No PCR products were detected by agarose gel electrophoresis, supporting the species-specificity of the candidate primer sets (DL, LC, and NL) in singleplex PCR assays and a multiplex assay (Table 3 and Supplementary Materials Figure S2).

**Figure 3:**
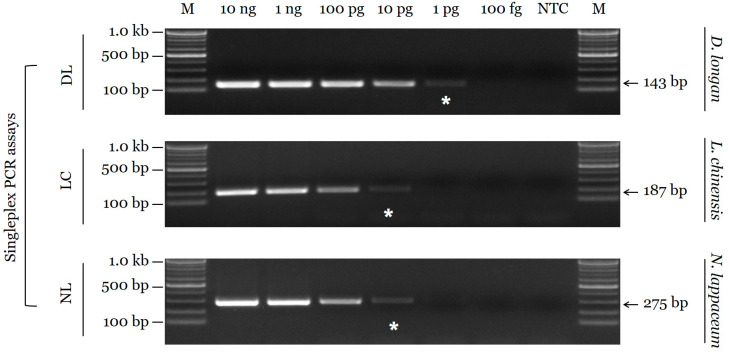
Sensitivity of singleplex PCR assays using the species-specific primer sets. M: 100 bp DNA size marker (100–1000 bp), NTC: no template control. Arrowheads indicate the amplified fragment sizes. White asterisks indicate the limit of detection.

**Figure 4:**
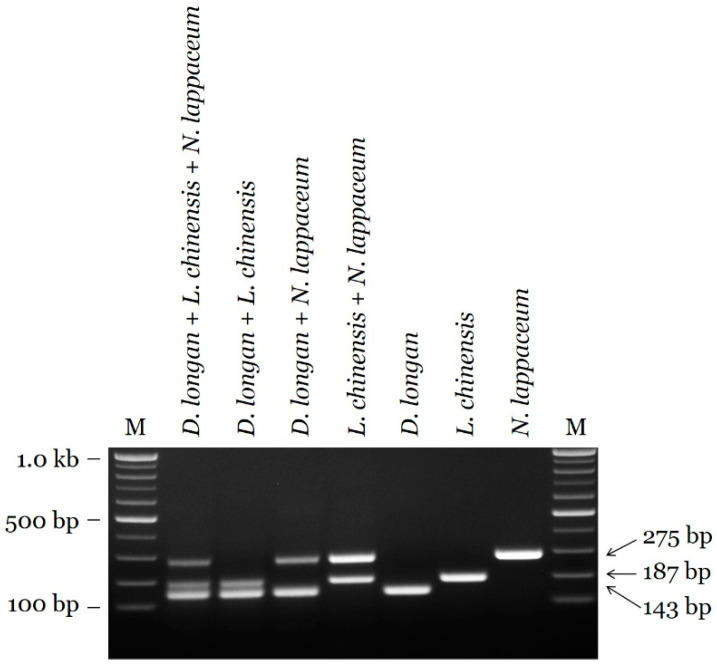
Discriminability in mixed samples of two or three species (*D. longan*, *L. chinensis*, and *N. lappaceum*) using the multiplex PCR assay. M: 100 bp DNA size marker (100–1000 bp). Arrowheads indicate the amplified fragment sizes.

The sensitivity of the candidate primer sets was evaluated by the limit of detection (LOD) using a 10-fold serially diluted template DNA in species-specific assays with three replicates. The LOD for the DL primer set was 1 pg, and those for the LC and NL primer sets were 10 pg (Figure 3). Sensitivity of the multiplex PCR assay was evaluated using a mixture of two or three species, and discriminability was clearly confirmed (Figure 4 and Supplementary Materials Figure S3).

### 2.4. Application to Commercial Products

**Figure 5:**
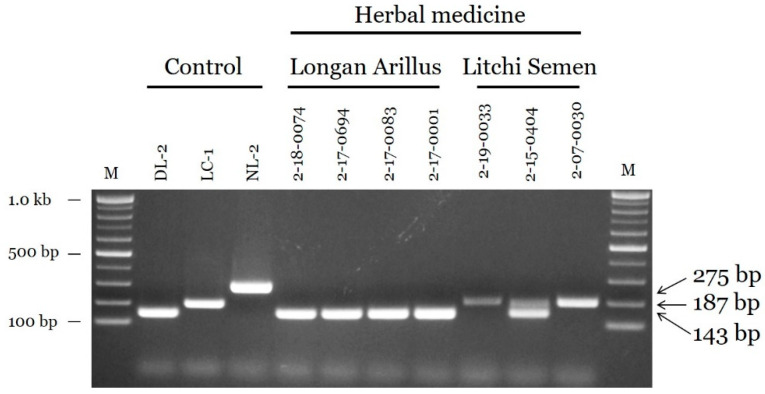
Monitoring of herbal medicines distributed as Longan Arillus and Litchi Semen using the multiplex PCR assay. M: 100 bp DNA size marker (100–1000 bp), DL-2: *D. longan*, LC-1: *L. chinensis*, NL-2: *N. lappaceum*. See the Supplementary Materials Table S1 for more detailed information about control samples. ‘Longan Arillus’ (voucher number: 2-18-0074, 2-17-0694, 2-17-0083, and 2-17-0001) and ‘Litchi Semen’ (voucher number: 2-19-0033, 2-15-0404, and 2-07-0030) are commercial products. Arrowheads indicate the amplified fragment sizes.

The newly developed multiplex PCR assay was used to analyze seven samples distributed as Longan Arillus and Litchi Semen. First, the seven samples were authenticated based on morphological characteristics by a herbologist. The four Longan Arillus samples were identified as authentic *D. longan* (Figure 5). Two of three Litchi Semen samples were identified as authentic *L. chinensis*, and one sample (voucher number: 2-15-0404) was identified as a mixture of *L. chinensis* and *D. longan* (Figure 5). PCR amplicons for adulterants 2-15-0404′ amplified by multiplex PCR were confirmed by Sanger sequencing, indicating that the sample contained both *D. longan* and *L. chinensis*.

## 3. Discussion

The adulteration of herbal medicine has negative effects on standardization and efficacy. Nevertheless, adulteration with species with similar morphologies frequently occurs for economic reasons. Several studies have evaluated adulterants in herbal medicine focused on the detection using molecular tools [8,14,15,17]. Longan Arillus and Litchi Semen have similar aril and seed morphologies but distinct uses [4]. In our phylogenetic analysis, we found that ITS sequences could clearly distinguish between *D. longan*, *L. chinensis*, and *N. lappaceum*, as well as closely related species belonging to the family Sapindaceae (Figure 1).

Based on ITS sequences, we developed a reliable and efficient assay for species identification. Interspecific distances for the target species-specific sequence region were high among the three species [8,14]. In addition, intraspecific distances for the conserved sequence region were low. Species-specific primers were designed based on inter- and intraspecific sequence variation, ensuring differential amplicon sizes for the multiplex PCR assay. Moreover, primers producing smaller PCR products (143 bp for *D. longan*, 189 bp for *L. chinensis*, and 276 bp for *N. lappaceum*) than the ITS barcoding sequences of 652–681 bp were advantageous for PCR amplification. As shown in Figure 2 and Figure 4, the specificity of candidate primers was validated in singleplex PCR assays and the multiplex PCR assay. The ITS region is found in all genomes, including those of plants, animals, fungi, and microorganisms [9,16]. An analysis of cross-reactivity using 23 species used as herbal medicines supported the specificity of the newly developed SCAR markers, as evidenced by the lack of amplification of closely related species or other taxa. Marker sensitivity is an important index for applications to herbal medicines, which are commonly distributed as slices or powders [15,16]. Unintended adulterants could be combined with authentic species during plant collection, and economically motivated adulteration is common. We confirmed that the SCAR markers exhibit high specificity, with limits of detection of 1 pg (*D. longan*) and 10 pg (*L. chinensis* and *N. lappaceum*). Moreover, we validated our assay by accurately detecting adulterants in commercial Litchi Semen. The species-specific SCAR marker is expected to contribute substantially to the standardization of the herbal medicines Longan Arillus and Litchi Semen.

## 4. Materials and Methods

### 4.1. Plant Material and DNA Extraction

Fifteen samples of *D. longan*, *L. chinensis*, or *N. lappaceum* were obtained. Four samples (KIOM201201005283, KIOM201201005284, KIOM201201005390, and KIOM201201005391) were collected by plant taxonomists, ecologists, and herbologists from different regions in China (Supplementary Materials Table S1). The other 11 samples were purchased from fruit distributors. The collected samples were deposited in the Korea Institute of Oriental Medicine (KIOM) herbarium (Index Herbariorum (IH) code: KIOM), and fresh leaves were stored at −75 °C. These voucher specimens were identified by the Classification and Identification Committee of the KIOM. Genomic DNA from 15 samples were extracted using the DNeasy Plant Mini Kit (Qiagen, Valencia, CA, USA) according to the manufacturer’s instructions. The DNA concentration was measured using the ND-1000 UV/Vis spectrophotometer (NanoDrop, Wilmington, DE, USA).

### 4.2. Sequence Statistics and Phylogenetic Analysis

The ITS region was amplified using ITS1 (5′-TCC GTA GGT GAA CCT GCG G-3′) and ITS4 (5′-TCC TCC GCT TAT TGA TAT GC-3′) primers [18]. PCRs were performed in a 40 µL reaction mixture containing 10 ng template, 0.5 µmol L^−1^ each primer, and SolgTM 2 × Taq PCR Smart-Mix I (Solgent, Daejeon, Korea). PCR conditions were as follows: initial denaturation at 95 °C for 2 min, 35 cycles of 95 °C for 1 min, 53 °C for 1 min, and 72 °C for 2 min, and a final elongation at 72 °C for 5 min. Subsequent experimental steps, such as gel rescue, T-vector subcloning, and sequence analyses, were performed as described previously [8]. ClustalW implemented in BioEdit version 7.2.5 was used to generate sequence alignments [19]. Sequence statistics and phylogenetic trees were analyzed using MEGAX version 10.1.7 [20].

### 4.3. Species-Specific/Multiplex SCAR Assay

Species-specific primers were designed using indels and nucleotide substitutions based on ITS sequences of 15 samples (*D. longan*, *L. chinensis*, and *N. lappaceum*) [8]. PCRs were performed in a 20 µL reaction mixture containing 10 ng template, 0.5 µmol L^−1^ each primer, and SolgTM 2 × Taq PCR Smart-Mix I (Solgent). PCR conditions were as follows: initial denaturation at 95 °C for 2 min, 33 cycles of 95 °C for 20 s, 63 °C for 30 s, and 72 °C for 30 s, with a final elongation at 72 °C for 5 min [8]. All experiments were verified with three replicates.

### 4.4. Specificity and Sensitivity

The theoretical specificity of the species-specific primers was analyzed using a multiple alignment generated using ClustalW against the ITS sequences registered in the GenBank database for Sapindaceae, which includes *D. longan*, *L. chinensis*, and *N. lappaceum* (Supplementary Materials Figure S4). The specificity of the SCAR primers was tested using 14 plant species, 8 animal species, and 1 fungal species used as herbal medicines. The sensitivities of singleplex PCR assays were analyzed using a 10-fold serially diluted template DNA (10 ng, 1 ng, 100 pg, 10 pg, and 100 fg) from each species. The sensitivities of multiplex PCR assay were analyzed using a 10-fold serially diluted template from the mixed two or three species. Experimental conditions used for singleplex PCR assays and the multiplex PCR assay were the same as those described above (Section 4.3). Sensitivity tests were verified with three replicates.

### 4.5. Monitoring of Longan Arillus and Litchi Semen

Seven herbal medicine samples (four Longan Arillus and three Litchi Semen) were used for monitoring. Each sample was prepared as a 10 g mixture using a grinder, and genomic DNA was extracted. Experimental conditions used for singleplex PCR assays and the multiplex PCR assay were the same as those described above (Section 4.3).

## 5. Conclusions

We analyzed the ITS sequences of three species, *D. longan* (longan), *L. chinensis* (litchi), and *N. lappaceum* (rambutan). Species-specific primer sets (i.e., SCAR markers) were validated for singleplex PCR assays and a multiplex PCR assay. The newly developed SCAR markers had high sensitivity and specificity. Furthermore, the application of the multiplex PCR assay to commercial herbal medicines supported its ability to effectively discriminate against Longan Arillus and Litchi Semen.

## Figures and Tables

**Table 1:** Summary statistics for ITS sequences.

Species	Amplicon Length (bp)	Intraspecific Distance	Interspecific Distance	G + C (%)
*Dimocarpus longan*	661	0.0009 ± 0.0034	0.0925 ± 0.0179	61.6
*Litchi chinensis*	652	0.0033 ± 0.0018	0.0880 ± 0.0130	64.0
*Nephelium lappaceum*	681	0.0111 ± 0.0094	0.1051 ± 0.0067	64.4

**Table 2:** Primer information for the singleplex and multiplex PCR assays.

SCAR Marker	Primer	Target Species	Sequence (5′–3′)	Size (bp)
DL	DL-F	*D. longan*	GCC TCC CGT GGG ACG CTT AA	143
	DL-R		TCA GGG TCG GGA GCC TTC AA	
LC	LC-F	*L. chinensis*	AGG CGT GGG GAT GCG TTA TC	189
	LC-R		GGT CGC GAT GCG TGA CGG T	
NL	NL-F	*N. lappaceum*	AAG TTG CGC CCC AAG CCG T	276
	NL-R		CGT CGG GAT CGC GAC GCT TC	

**Table 3:** Specificity of singleplex and multiplex PCR assays based on cross-reactivity with plant, animal, and fungal taxa used as herbal medicines.

Species	Herbal Name	Singleplex Assays			Multiplex Assay (DL + LC + NL)
		DL	LC	NL	
*Dimocarpus longan*	Longan Arillus	+	-	-	+(DL)
*Litchi chinensis*	Litchi Semen	-	+	-	+(LC)
*Nephelium lappaceum*	*No name*	-	-	+	+(NL)
*Schisandra chinensis*	Schisandrae Fructus	-	-	-	-
*Zanthoxylum schinifolium*	Zanthoxyli Pericarpium	-	-	-	-
*Aralia continentalis*	Araliae Continentalis Radix	-	-	-	-
*Cynanchum wilfordii*	Cynanchi Wilfordii Radix	-	-	-	-
*Angelica dahurica*	Angelicae Dahuricae Radix	-	-	-	-
*Paeonia lactiflora*	Paeoniae Radix	-	-	-	-
*Akebia quinata*	Akebiae Caulis	-	-	-	-
*Viscum coloratum*	Visci Ramulus et Folium	-	-	-	-
*Glehnia littoralis*	Glehniae Radix	-	-	-	-
*Acorus gramineus*	Acori Graminei Rhizoma	-	-	-	-
*Paeonia × suffruticosa*	Moutan Radicis Cortex	-	-	-	-
*Sigesbeckia orientalis* subsp. *pubescens*	Siegesbeckiae Herba	-	-	-	-
*Rheum rhabarbarum*	Rhei Undulatai Rhizoma	-	-	-	-
*Machilus thunbergii*	Machilii thunbergii cortex	-	-	-	-
*Scolopendra subspinipes*	Scolopendra	-	-	-	-
*Metaphire guillelmi*	Lumbricus	-	-	-	-
*Cryptotympana atrata*	Cicadidae Periostracum	-	-	-	-
*Gekko gecko*	Gecko	-	-	-	-
*Bombyx mori*	Batryticatus Bombyx	-	-	-	-
*Pelodiscus sinensis*	Pelodiscis Carapax	-	-	-	-
*Hippocampus trimaculatus*	Hippocampus	-	-	-	-
*Elaphe carinata*	Serpentis Periostracum	-	-	-	-
*Ophiocordyceps sinensis*	Cordyceps	-	-	-	-

+: fragments detected by species-specific SCAR markers, -: not detected.

## Supplementary Materials

The following are available online at https://www.mdpi.com/2223-7747/9/8/948/s1, Figure S1: ClustalW multiple alignment of internal transcribed spacer (ITS) sequences from *D. longan*, *L. chinensis*, and *N. lappaceum*, Figure S2: Specificity of singleplex PCR assays and a multiplex PCR assay based on plant, animal, and fungal species used as herbal medicines. M: 100 bp DNA size marker (100–1000 bp), Figure S3: Sensitivity of multiplex PCR assays using the species-specific primer sets. M: 100 bp DNA size marker (100–500 bp), Figure S4: ClustalW multiple alignment of internal transcribed spacer (ITS) sequences in 12 species belonging to the family Sapindaceae, Table S1: Plant material and related information, Table S2: Plant, animal, and fungal species used to evaluate the specificity of singleplex PCR assays and the multiplex PCR assay.
