# Supplementary figures and images for: Accurate and Rapid Identification of Longan Arillus and Litchi Semen by a Multiplex PCR Assay
Source: Plants (Basel). 2020 Jul 28;9(8):948. doi: 10.3390/plants9080948 (PMC7464683; doi:10.3390/plants9080948)

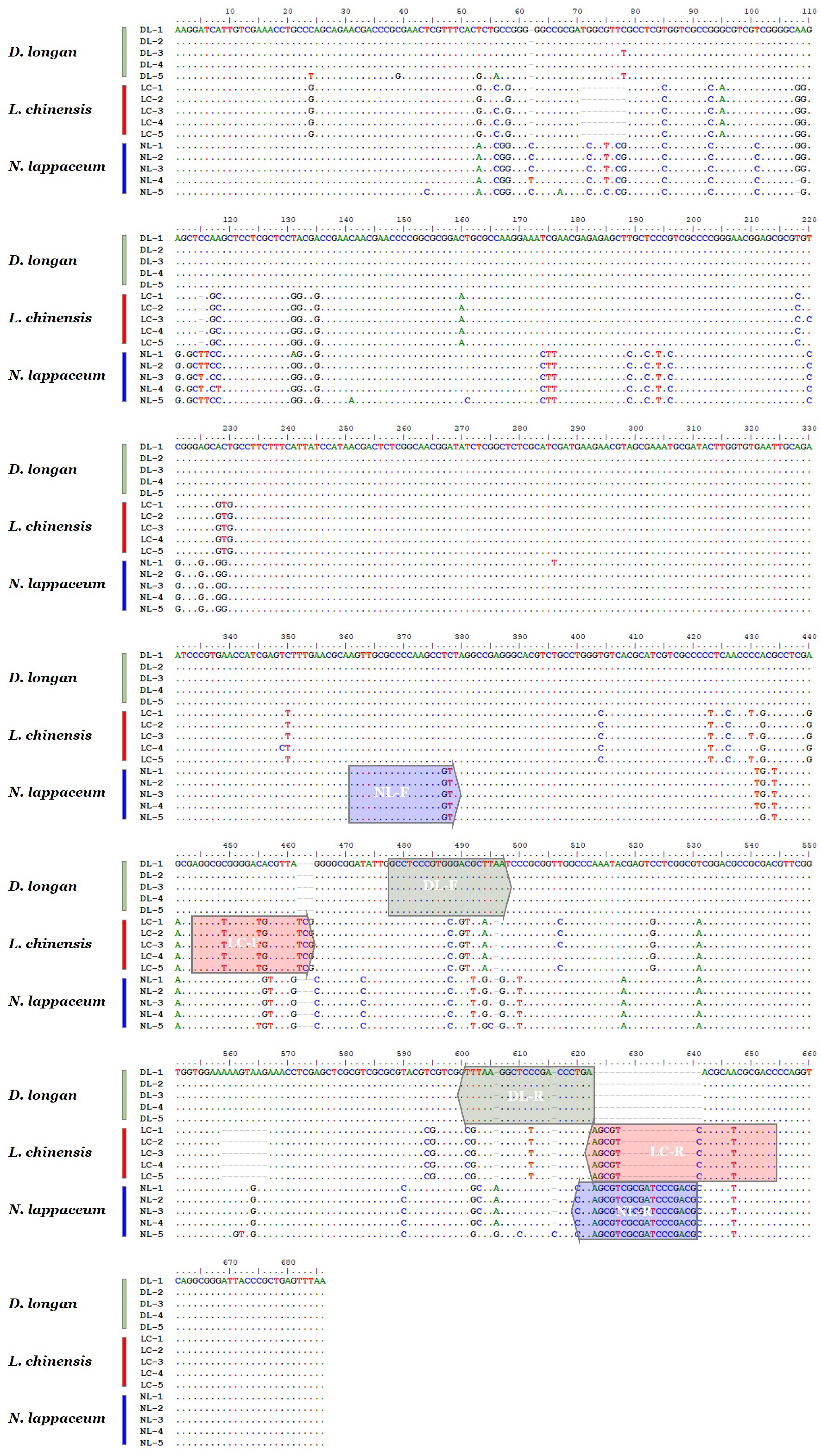

Supplement: Supplementary file 1 [file plants-09-00948-s001.zip › plants-875591-supplementary-proof/Supplementary Figure S1.jpg]

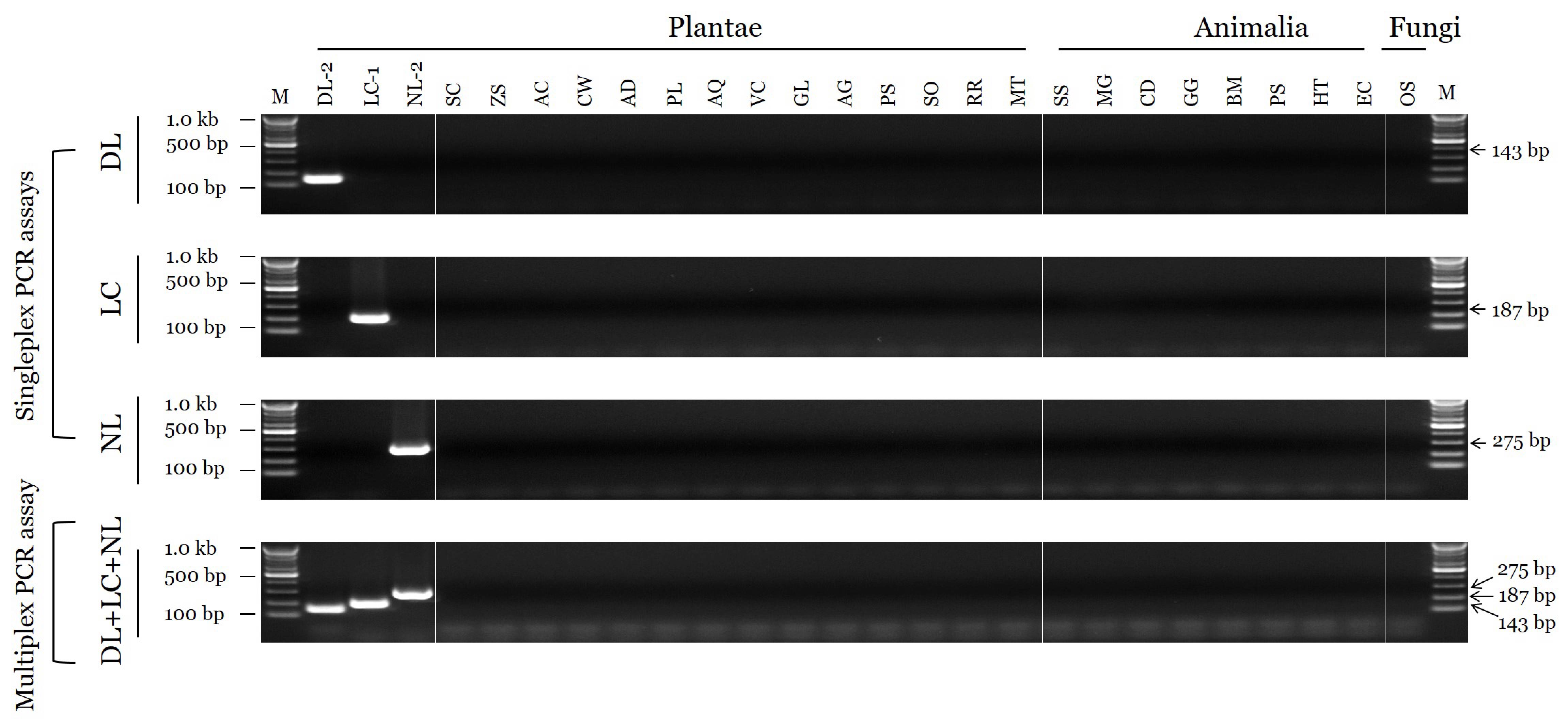

Supplement: Supplementary file 1 [file plants-09-00948-s001.zip › plants-875591-supplementary-proof/Supplementary Figure S2.jpg]

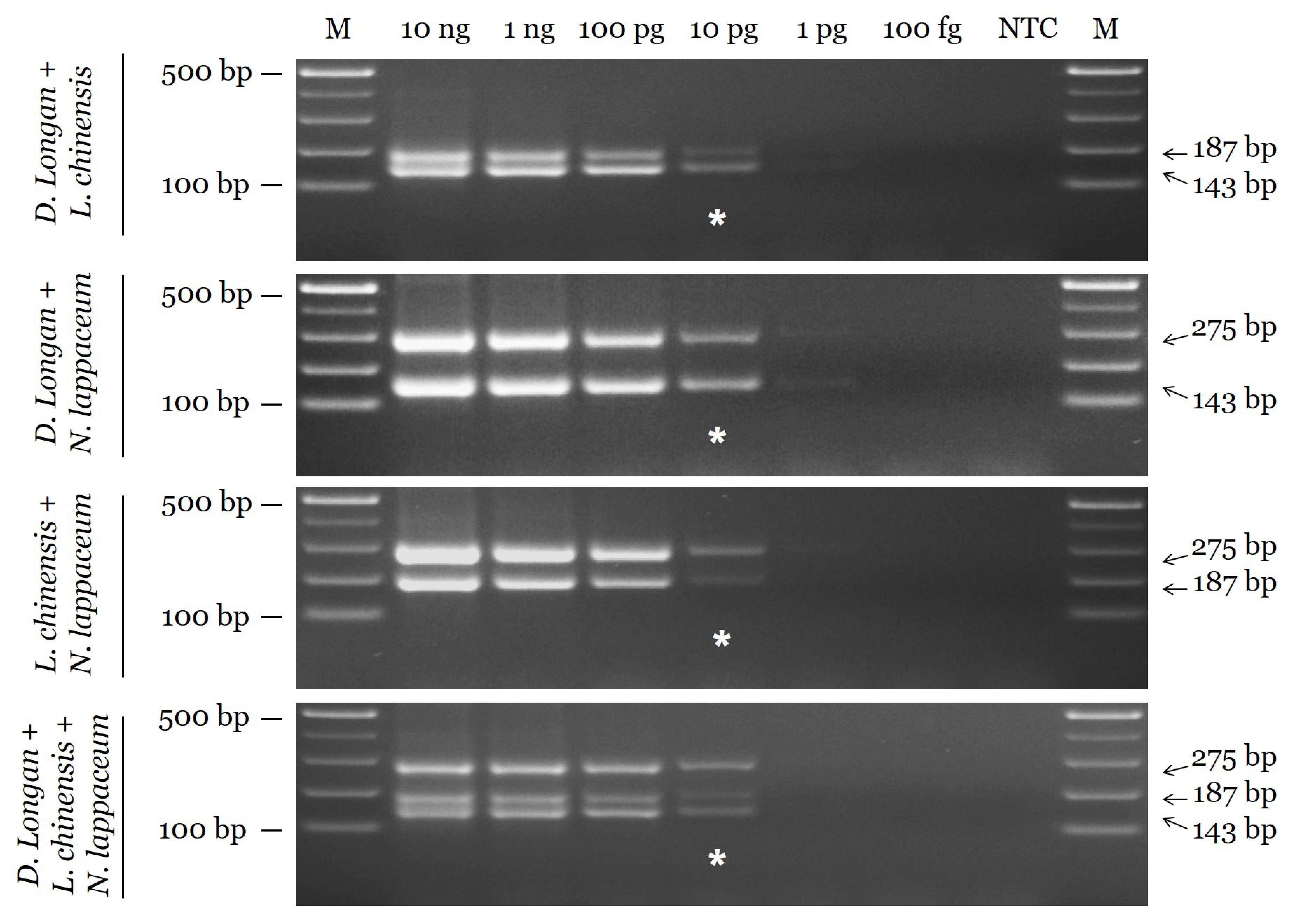

Supplement: Supplementary file 1 [file plants-09-00948-s001.zip › plants-875591-supplementary-proof/Supplementary Figure S3.jpg]

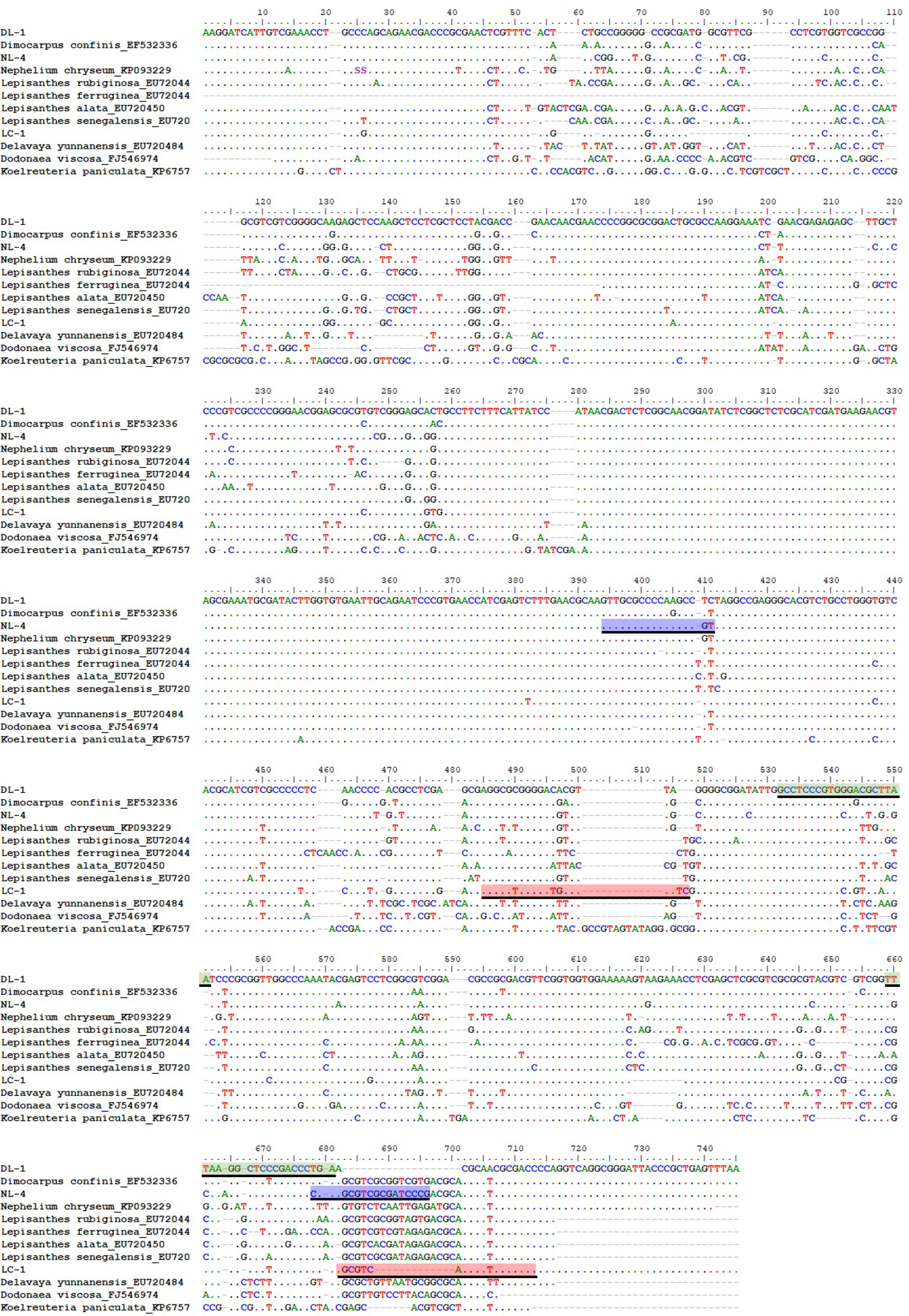

Supplement: Supplementary file 1 [file plants-09-00948-s001.zip › plants-875591-supplementary-proof/Supplementary Figure S4.jpg]
